# Quantitative morphological analysis of 2D images of complex-shaped branching biological growth forms: the example of branching thalli of liverworts

**DOI:** 10.1186/s13104-017-2424-0

**Published:** 2017-02-20

**Authors:** Pirom Konglerd, Catherine Reeb, Fredrik Jansson, Jaap A. Kaandorp

**Affiliations:** 1 Computational Science Lab, University of Amsterdam, Amsterdam, The Netherlands; 2 Institut de Systématique Évolution et Biodiversité UMR7205, UPMC-MNHN-CNRS-EPHE, 57 Rue Cuvier, BP39, 75005 Paris, France

**Keywords:** Complex-shaped branching forms, Image analysis, Quantitative morphological analysis, Morphological variable, Liverworts

## Abstract

**Background:**

Many organisms such as plants can be characterized as complex-shaped branching forms. The morphological quantification of the forms is a support for a number of areas such as the effects of environmental factors and species discrimination. To date, there is no software package suitable for our dataset representing the forms. We therefore formulate methods for extracting morphological measurements from images of the forms.

**Results:**

As a case study we analyze two-dimensional images of samples from four groups belonging to three species of thalloid liverworts, genus *Riccardia*. The images are pre-processed and converted into binary images, then skeletonized to obtain a skeleton image, in which features such as junctions and terminals are detected. Morphological measurements known to characterize and discriminate the species in the samples such as junction thickness, branch thickness, terminal thickness, branch length, branch angle, and terminal spacing are then quantified. The measurements are used to distinguish among the four groups of *Riccardia* and also between the two groups of *Riccardia amazonica* collected in different locations, Africa and South America. Canonical discriminant analysis results show that those measurements are able to discriminate among the four groups. Additionally, it is able to discriminate *R. amazonica* collected in Africa from those collected in South America.

**Conclusions:**

This paper presents general automated methods implemented in our software for quantifying two-dimensional images of complex branching forms. The methods are used to compute a series of morphological measurements. We found significant results to distinguish *Riccardia* species by using the measurements. The methods are also applicable for analyzing other branching organisms. Our software is freely available under the GNU GPL.

**Electronic supplementary material:**

The online version of this article (doi:10.1186/s13104-017-2424-0) contains supplementary material, which is available to authorized users.

## Background

Quantification of morphological characteristics of biological objects has continuously posed a challenge due to varieties in their morphological changes. The morphological variation offers channels for numerous studies, for example, the comprehension of causes, factors, and directions of biological processes [1], the influence of environmental changes [2, 3]. Besides, it is used in taxonomy to identify, describe, and classify species or taxa as well as evolutionary systematics study. The growth morphology of many modular organisms such as plants presents a branched and complex-shaped structure. Their growth can be indeterminate [4], making the quantification and analysis of their form more complicated.

There are three well known morphometric approaches for form analysis: traditional, landmark-based, and outline-based. Landmark-based morphometrics [5] considers discrete anatomical loci, while outline-based captures outlines of form structures. Both are more suitable for non-modular organisms, but less applicable for the analysis of indeterminate growth forms of modular organisms, which prefers traditional approach by measuring linear distances (width, length), angles, and ratios.

There are several 2D and 3D imaging technologies used to gather quantitative characters related to the growth form. Although advances of 3D imaging techniques can theoretically quantify the characters more accurately, for some plant organisms such as liverworts, which are used in our study, their characteristic features are thin and flat; therefore, 2D imaging techniques are more suitable than 3D which are complicated in terms of procedures, implementation, and executing time. Moreover, in case of field work, it is more practical for 2D image acquisition by using a camera or a microscope.

Methods in morphological analysis of 2D images of the growth forms of branching organisms have been developed in several studies [6, 7]. In these studies the analysis is based on the construction of a 2D morphological skeleton of a branching object in the images. The morphological skeleton of the branching object can be used to measure various biologically relevant characteristics. Many steps in this analysis have been done in a manual way requiring visual interaction in many places. To date, there are open 2D image analysis software that perform on plant organs such as leaves (LeafJ [8], LAMINA [9], and leaf processor [10]). These programs can measure mainly shape, length, width, perimeter, surface of leaves, and leaf venation pattern, but they are not designed to measure some other important morphological traits, which are very essential to our samples, such as branch thickness and branch spacing. Root system architecture (RSA) software (DART [11], root system analyzer [12], RootReader2D [13]) are the most similar to our software. Their algorithms share some similar features such as junction radius, branch length, and branch angle; however, those also lack the measurements of branch thickness and branch spacing.

*Riccardia*, a plant genus in the liverwort family Aneuraceae, is represented by pinnate to tripinnate thalloid plants creeping or erecting on various substrates (rocks, dead wood, soil), and grows mainly in tropical areas and always in humid climate. Its dimension ranges from some millimeters to a few centimeters. It is the largest genus among the family Aneuraceae, with more than 300 names [14], which should be a hundred of accepted species after greatly-needed revision [15]. The genus has mystified bryologists for many years due to its polymorphic morphology. For taxonomical studies, the species are still doubtful in taxonomic classification and the morphological variability of *Riccardia* across its large geographical range has not been extensively studied, in particular, African *Riccardia.* In the literature, African *Riccardia* are described mostly by their morphological characters such as axis width, length, and angles [16–23], while other characters can also be expressed, for example, *Riccardia amazonica* is described as winged (wing is at least 2 rows of unistratose cells at the margin of the thallus) [23, 24] and as not winged [18, 19]. In any case, due to such plastic phenotypes, it is not easy to express their variability.

The aim in this study is to develop a general and semi-automatic software implementing methods to quantitatively measure and analyze morphological characters from a class of 2D image of complex-shaped branching objects stemming from indeterminate growth. The morphological characters are junction thickness, branch thickness, terminal thickness, branch length, branch angle, and terminal spacing. The methods are developed in the context of a review of African (and Indian Ocean) *Riccardia* which have never really been studied at the continental scale nor in an integrative framework. The morphometrical approach presented here will be used at a larger scale in order to be compared with molecular species delineation [25].

## Methods

### Plant materials

**Fig. 1:**
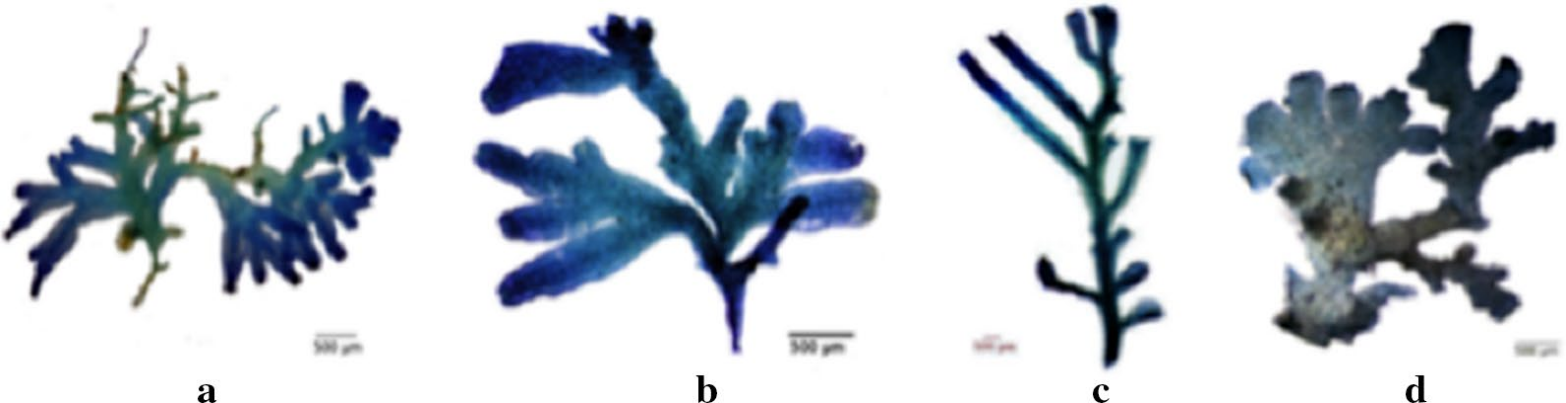
Sample images of thalloid liverwort *Riccardia,* Aneuraceae with different species, **a** *Riccardia amazonica*-AF, **b** *Riccardia amazonica*-SA, **c** *Riccardia compacta,* **d** *Riccardia obtusa*

*Riccardia* samples come from three recognized species [26]: *R. amazonica* (Spruce) Schiffn. exGradst., *Riccardia obtusa* S.W. Arnell, and *Riccardia compacta* (Steph.) Arnell. The samples were loaned by different herbaria (Additional file 1: Table SI1). As *Riccardia* collections are usually very intricate mats of plants, in several layers, each sample (collection pocket) contains dozen to hundreds of thalli. For each collection, 1–16 thalli were randomly picked with broken ones discarded in order to keep a natural variety of the complete living thalli. No recent field collections were conducted for this study and none of the species studied belongs to a protected species or to the convention on international trade in endangered species of wild fauna and flora (CITES). Since *R. amazonica* samples were collected from South America and Africa, we then separated the samples into *R. amazonica*-AF and *R. amazonica*-SA respectively. A total of 138 samples are therefore categorized into four taxonomical groups [Fig. 1, the number of the samples for each species is in (Additional file 1: Table SI2 )].

### Framework

**Fig. 2:**
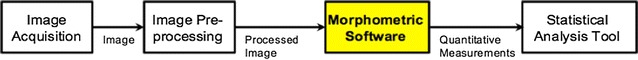
The framework of the morphological measurement and analysis system

Our framework consists of different procedures (Fig. 2). Images from Image Acquisition are pre-processed before Morphometric Software, which automatically produces quantitative measurements. A statistical analysis tool, R in our case, is then used to analyze the measurements.

### Image acquisition

The experimental images used in this work originate from two sources: (1) artificial images, which are used to systematically test the software (2) images of our samples. Each sample was precisely laid on a glass slide in a droplet of water, and their images were then taken with a Nikon Coolpix P6000 through a binocular microscope.

### Image pre-processing

We used the raster graphics editor, GIMP 2.6.12 [27] and the image processing package based on ImageJ, Fiji [28], to process original color sample images. The color image is converted to 8-bit grayscale, thereafter thresholding is performed using the Ostu method to obtain a binary image. However, the given threshold value is then adjusted to obtain the best binary image closet to the original image. Therefore, different threshold values are assigned for different images. We use morphological operations to improve the quality of the image by using opening operation to remove some stray foreground pixels in the background and closing operation to fill holes in the foreground.

### Morphometric software

**Fig. 3:**
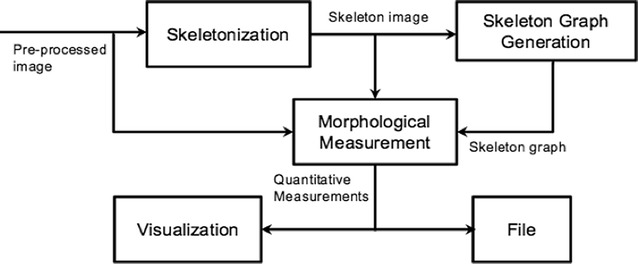
Schematic overview of procedures in our proposed software

In our morphometric software, a number of procedures were applied to obtain quantitative measurements (Fig. 3). Skeletonization produces a skeleton image from the pre-processed image. Skeleton graph generation then uses the skeleton image to generate skeleton graph. The morphological measurements use the pre-processed image, skeleton image and skeleton graph to produce a set of quantitative results, which are visualized in graphical representation and stored in text files.

**Fig. 4:**
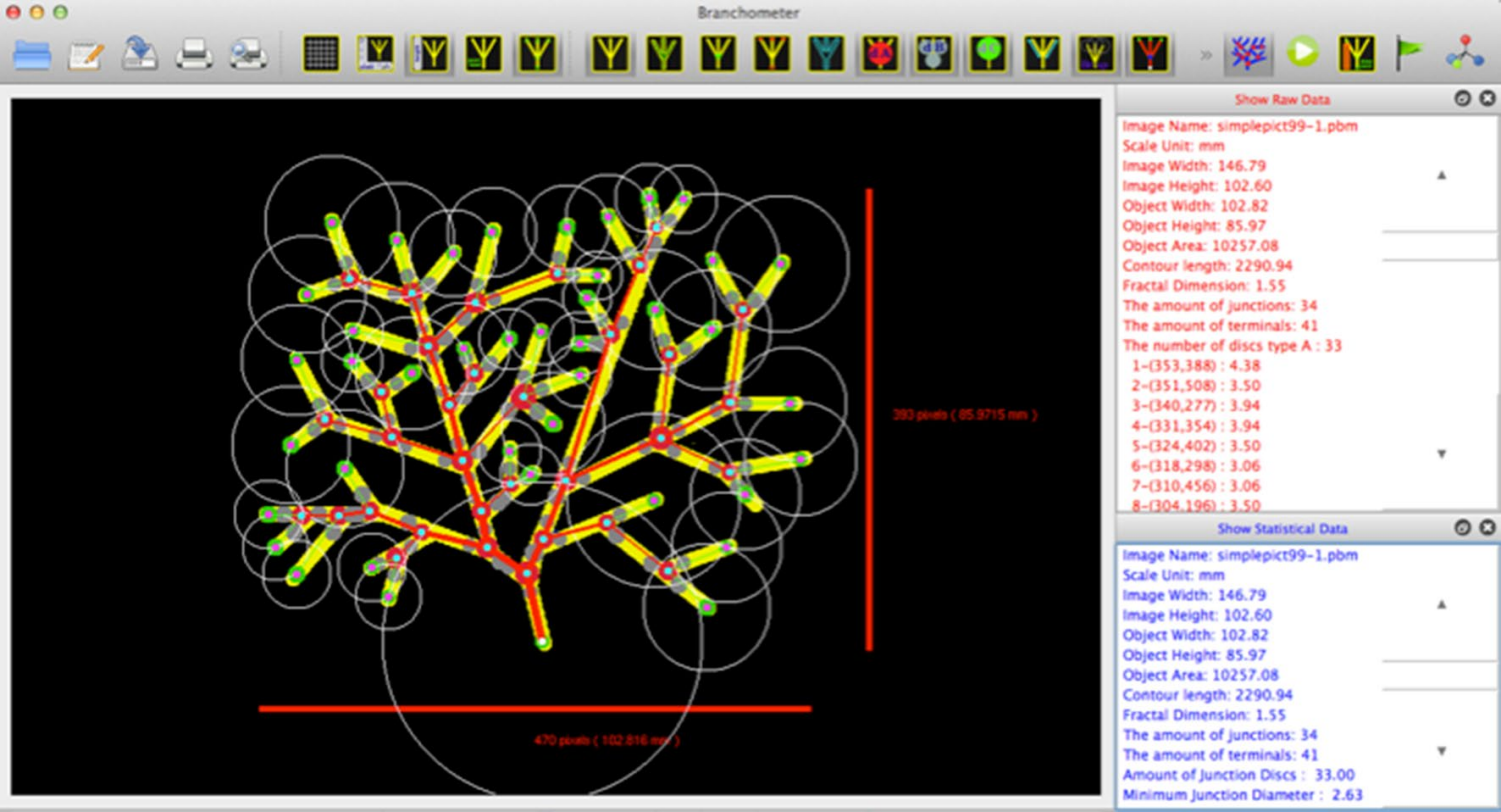
A screenshot of the graphical user interface of our software

The software was written entirely in C language for high performance under the developing environment of QT Creator. This allows for greater visual interactivity and control. The software is able to run on Mac and OSX, Linux, and Windows. The software is available under the GNU General Public License (GNU GPL), and can be downloaded from https://github.com/UvA-compsci/branchometer. Figure 4 shows its graphical user interface.

### Skeletonization

Skeletonization is the process of reducing an image to its skeleton. By reducing an object to only a skeleton, unimportant features and image noise are filtered out. Additionally, it is easier to determine critical features such as connection points and end points as well as greatly speeding up any subsequent analysis of images. Skeletonization algorithms are generally classified into three categories: (1) distance transforming method, which converts the original image into foreground and background elements, generates a distance map where each element gives the distance to the nearest background element and then detects ridges in the distance map as skeletal points. This method guarantees a central position of the skeleton, but it is sensitive to the noise, and generally doesn’t guarantee the skeleton connectivity [29, 30]. (2) Voronoi-Skeleton method, which calculates the Voronoi diagram generated by the boundary points or pixels. When the number of boundary points goes to infinity, the corresponding diagram converges to the skeleton [31]. It generates a connected skeleton, however it is a time consuming process especially for large objects. Therefore, it is not suitable to be applied complicated images to branching objects used in our study. (3) Thinning methods, which remove the pixels from the object boundary that will not change the topology of the object until obtaining a single-pixel-wide skeleton. Thus the method preserves the topology and connectivity of the skeleton, and guarantees the medial position of the skeleton [32, 33].

For our purpose, we needed a method that guaranteed the connectivity and topology of the skeleton in order to form the skeleton graph; therefore the thinning method was adopted. There are many thinning algorithms available such as the Zhang Suen algorithm [34], the Rosenfeld algorithm [35] and the Hilditch algorithm [36]. The skeletonization in this study was based on the thinning algorithm by Zhang and Suen because it is robust, fast, and easy to implement. The algorithm uses a lookup table to repeatedly remove pixels from the edges of objects in a binary image until a single-pixel-wide skeleton remains. After the skeletonization is done, the following significant features are extracted: (1) junction, a point having three or more adjacent points (branches) in a skeleton. (2) Terminal, the endpoint or tip of the skeleton.

### Skeleton graph generation

**Fig. 5:**
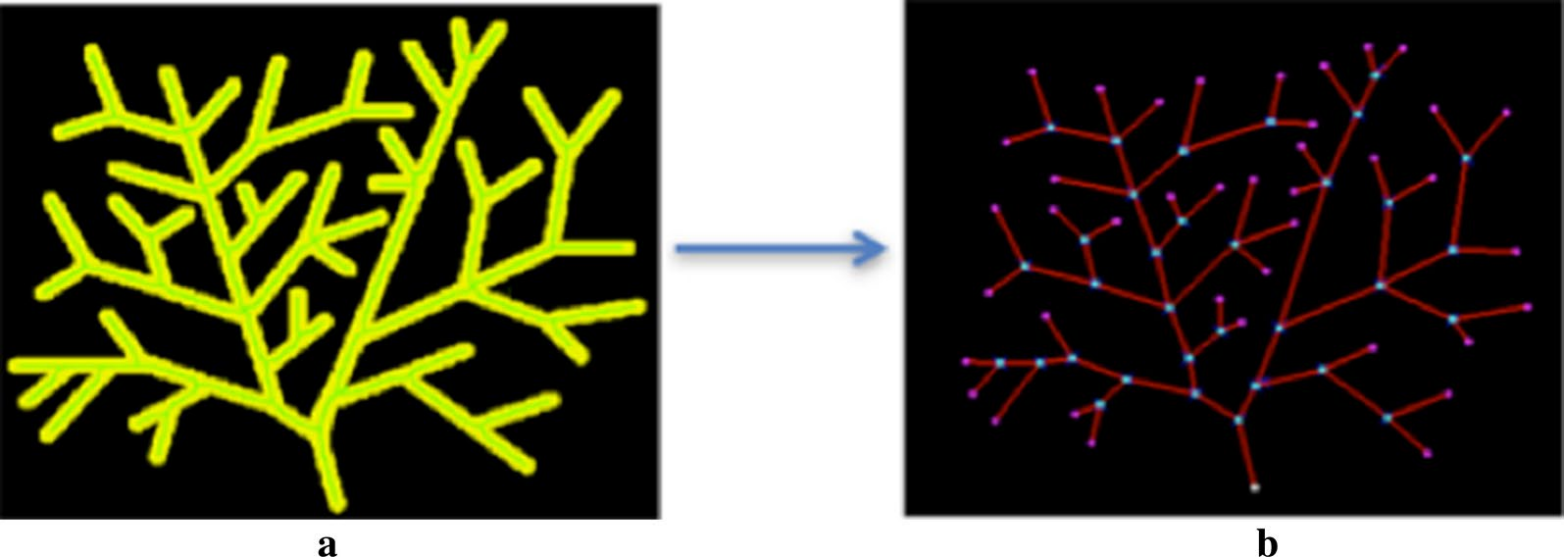
Graph representation of a skeleton. **a** Object image (*yellow*) with its skeleton (*green line*), **b** the generated graph from the skeleton in **a** shows *light blue dots* as junctions, *pink dots* as terminals, a *white dot* as the root of the skeleton graph, and *red lines* as links among junctions and terminals

Graph representation, which only preserves the topological structure of the object, is an essential step for the image measurement and analysis. The measurement and analysis can substantially be simplified if the skeleton image is represented as a formal graph structure. The graph is defined as G = (V, E) where V is a set of vertices or nodes, E is a set of edges or curves connecting the vertices. In this case, V is junctions or terminals and E is branches in the skeleton (Fig. 5). By following the skeleton pixels in the 8-connectivity sense from a vertex to another vertex, each branch path and length will be kept. To travel through the graph, depth first search algorithm is used by starting from a terminal selected as a root of the graph and keep track of all visited junctions, terminals and branch paths.

### Morphological measurements

We used morphometric methods to automatically quantify a number of localized morphological variables. These variables are thought to be useful in various applications, for instance, growth study that tells branch splitting rate, environmental influences on growth, and species classification that uses them as continuous characters to differentiate species. The variables are further used to discriminate species among the genus *Riccardia* as our case study. The measurement results are initially calculated in pixels. A scale tool provided by our software allows the user to define the pixel to other unit scale and all the measurements will be calculated from the scale setting.

#### Junction thickness (da)

**Fig. 6:**
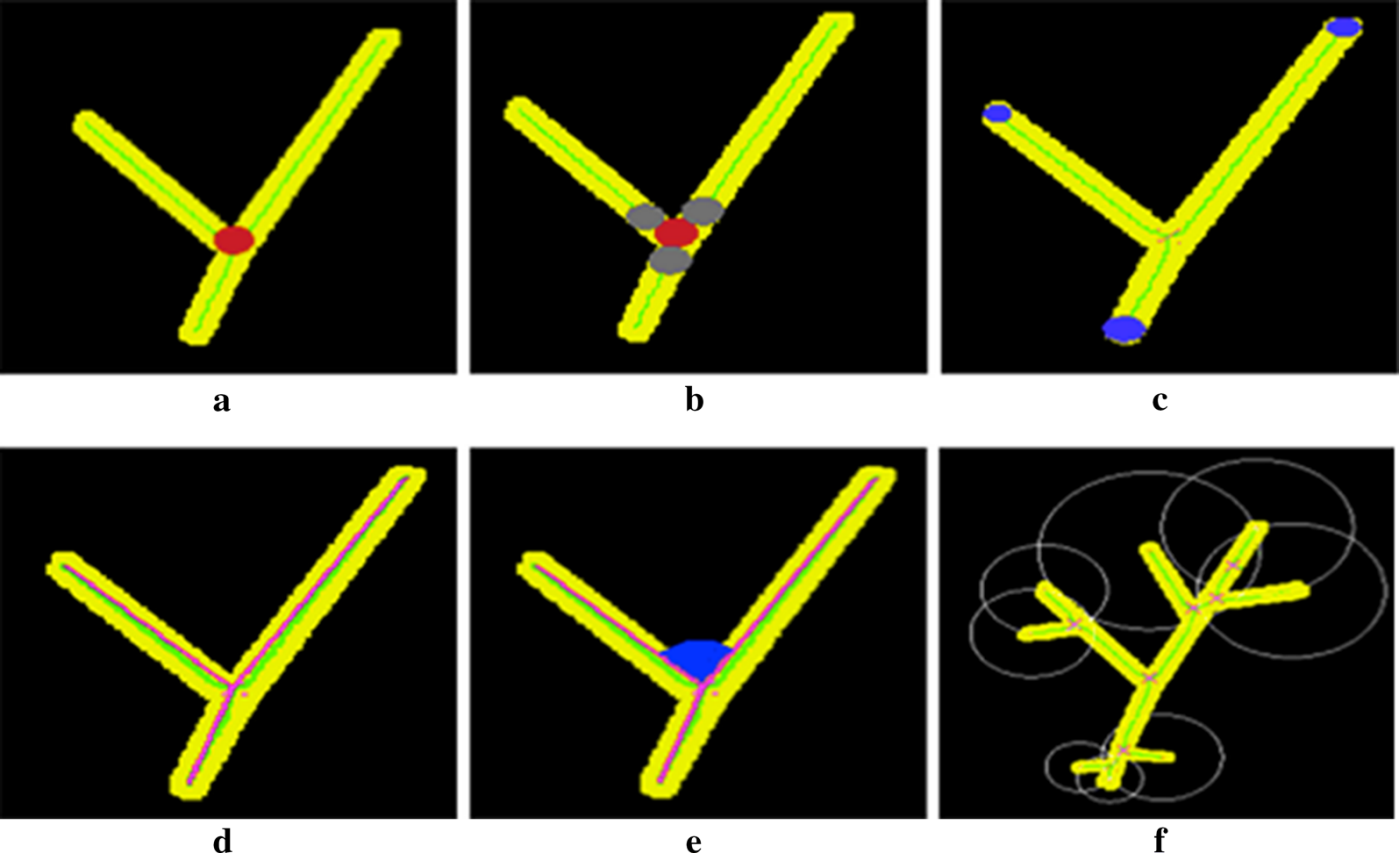
Types of measurements, the object image is shown in *yellow color* with skeleton in *green line*. **a** Junction thickness (*red disc*), **b** branch thickness (*gray disc*), **c**) terminal thickness (*blue disc*), **d** branch length (*pink line*), **e** branch angle (*blue area*), **f** branch spacing (*white circle*)

The thickness of the branch centered at a junction in the skeleton. The circular disc (Fig. 6a) representing da is created by using euclidean distance map [37] which calculates the shortest euclidean distance from the junction to the background of the image.

#### Branch thickness (db)

The thickness of the branch centered on a point along the skeleton after its last found da. The circular disc (Fig. 6b) representing db is created by using the euclidean distance map which calculates the shortest distance from a point along the skeleton to the boundary of the da or the background of the image.

#### Terminal thickness (dc)

The thickness of the terminal branch centered at the tip of the skeleton. Similar to da, the circular disc (Fig. 6c) representing dc is created by using the euclidean distance map which calculates the shortest distance to the background of the image.

#### Branch length

The number of branch pixels along the skeleton and euclidean distance between two successive vertices (Fig. 6d).

#### Branch angle

The angle between the two vectors originating from a junction in the skeleton and the center of successive db disc (Fig. 6e).

#### Branch spacing

The euclidean distance from a tip of a terminal branch to the nearest tip of another terminal branch (Fig. 6f).

### Statistical analysis

The morphological quantification results obtained by our software are analyzed with analysis of variance (ANOVA) to confirm the significant differences among the four taxonomical groups of *Riccardia* by taking one morphological variable at a time. Multivariate analysis of variance (MANOVA) assesses the statistical significance of the group differences by considering all of the variables simultaneously. Our analysis goal is to distinguish a group from the four groups by considering the variables; therefore, canonical discriminant analysis (CDA) was applied by finding the combinations of the variables that maximize the discrimination of the predefined groups, testing whether the means of those groups are significantly different, and computing classification rate. Statistical analysis was performed using RStudio Team [38].

## Results

We used a dataset with 138 images from the four taxonomical groups of thalloid liverworts, which consist of 37 samples from *R. amazonica* collected from Africa, 26 samples of *R. amazonica* collected from South America, 25 samples of *R. compacta* collected from Africa, and 50 samples of *Riccardia obtusa* collected from Africa. The morphological variables described above were quantified with our software. The graphical result of some of the sample images of thalloid liverworts are shown in Additional file 2: Figure SI1. Table 1 shows descriptive statistics of the six morphological measurement variables for each group.

**Table 1:** Descriptive statistics of the measured morphological variables according to the four groups of samples

Morphological variable	Mean ± SD			
	*R. amazonica_*AF N = 37	*R. amazonica* **_**SA N = 26	*R. compacta* N = 25	*R. obtusa* N = 50
Junction thickness	0.4 ± 0.09	0.49 ± 0.14	0.31 ± 0.05	0.67 ± 0.06
Branch thickness	0.28 ± 0.07	0.34 ± 0.08	0.22 ± 0.04	0.45 ± 0.11
Terminal thickness	0.18 ± 0.05	0.22 ± 0.07	0.15 ± 0.03	0.29 ± 0.07
Branch length	1.03 ± 0.25	1.31 ± 0.35	1.47 ± 0.46	1.28 ± 0.28
Branch spacing	0.99 ± 0.31	1.33 ± 0.3	1.33 ± 0.43	1.18 ± 0.31
Branch angle	119.02 ± 15.94	118.88 ± 18.02	128.19 ± 14.74	112.69 ± 14.74

**Fig. 7:**
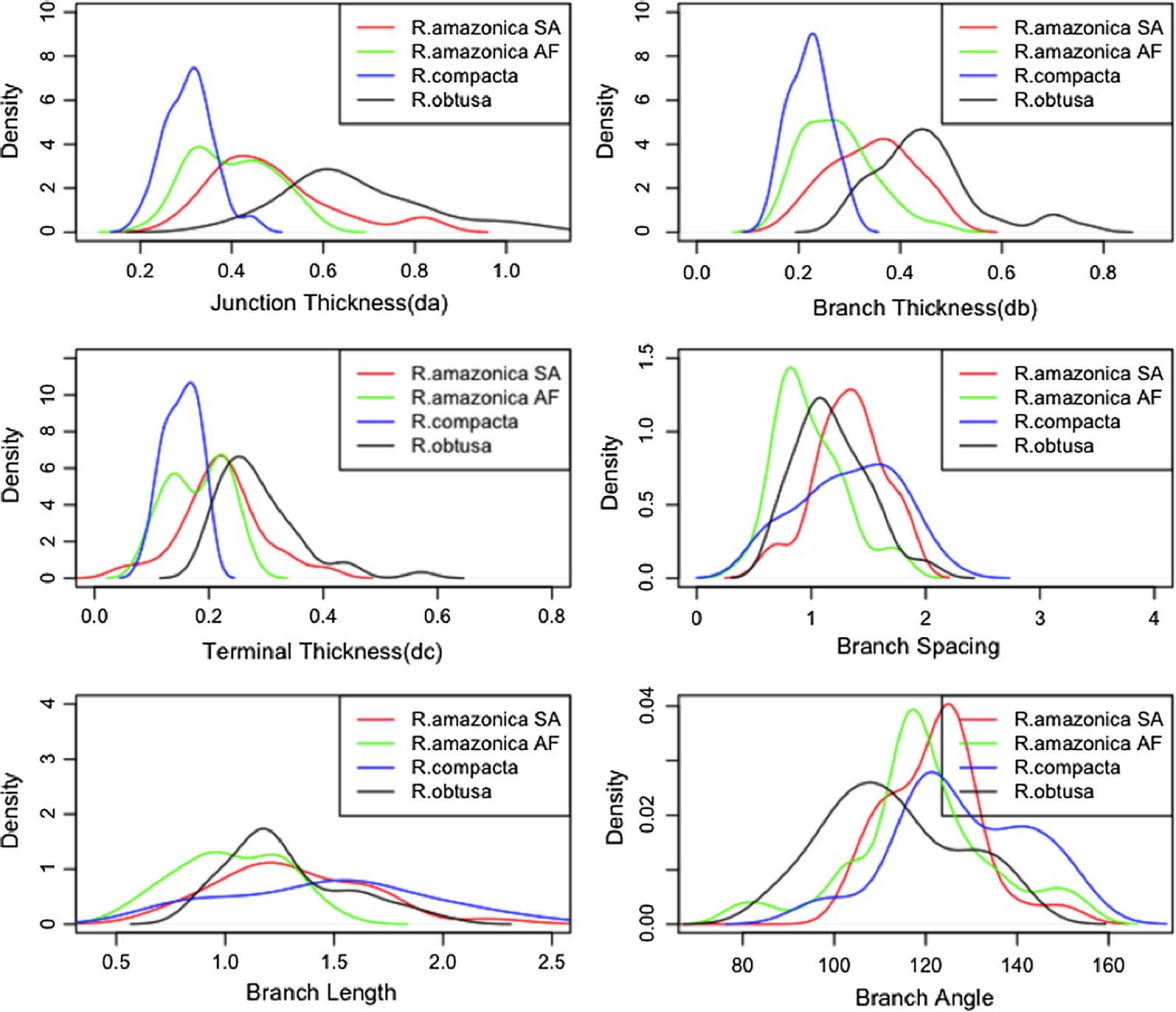
The density plot of taxonomical groups of *Riccardia* with the six measured morphological variables: junction thickness, branch thickness, terminal thickness, branch length, branch spacing, and branch angle

From Table 1a, means of some variables seem to differ noticeably among the four groups, which offer an opportunity to use the variables to distinguish samples in one group from the others. As considering the variables for *R. amazonica* samples from Africa (*R. amazonica*_AF), the mean value of its branch thickness, terminal thickness, branch length and branch spacing is lower than those from South America (*R. amazonica*_SA) except junction thickness which is very close each other. Therefore, it is possible to consider branch thickness, terminal thickness, branch length and branch spacing to separate the two groups. Moreover, *R. compacta* has the lowest mean value while *R. obtusa* has the highest mean value for junction thickness, branch thickness, and terminal thickness. The density plot of the mean of the morphological variables (Fig. 7) is also demonstrated and the Shapiro-Wilks normality test (Additional file 1: Table SI3) indicates that the variables are likely normally distributed.

From Fig. 7, the distribution of the mean values of each of the six morphological variables among the four groups appears that they differ from one another, except branch length and the branch angle between *R. amazonica* SA and *R. compacta*. Also, the result of ANOVA reveals significant differences among the four groups by considering each of the variables (Additional file 1: Table SI4).

Univariate ANOVA by using multiple comparison method was conducted to test the significant differences of each morphological variables. For each pair of the four groups, we test whether the mean of morphological variables are significantly different from each other. From the result of *p* value in Table 2, *p* value of the junction thickness (da) for each pair of the four groups is significant (*p* value <0.0001). This means junction thickness can differentiate among those groups, whereas *p* values of branch angle (ba) are insignificant for all pairs of the four groups except the pair between *R. obtusa* (R.ob) and *R. compacta* (R.co). Additionally, as considering *p* value of da, db, and dc, it is possible to take the combination of da, db, and dc into an account to separate the four groups. However this univariate approach does not account for correlation among the variables.

**Table 2:** *p* values of six morphological variables for each pair of the four groups using ANOVA

Morphological variable	*p* value					
	R.a.AF vs R.a.SA	R.a. AF vs R.ob	R.a.AF vs R.co	R.a.SA vs R.ob	R.a.SA vs R.co	R.ob vs R.co
da	0.00039 (**)	<0.0001 (***)	<0.0001 (***)	<0.0001 (***)	<0.0001 (***)	<0.0001 (***)
db	0.00078 (***)	<0.0001 (***)	0.00077 (***)	<0.0001 (***)	<0.0001 (***)	<0.0001 (***)
dc	0.00087 (**)	<0.0001 (***)	0.0146 (*)	0.00069 (***)	<0.0001 (***)	<0.0001 (***)
bl	0.00031 (***)	<0.0001 (***)	<0.0001 (***)	0.69 (ns)	0.18(ns)	0.036 (*)
bs	<0.0001 (***)	0.0062 (**)	0.00051 (***)	0.042 (*)	0.99(ns)	0.8 (.)
ba	0.973 (ns)	0.059 (.)	0.0257 (*)	0.112 (ns)	0.049 (*)	<0.0001 (***)

*R.a.AF* Riccardia amazonica collected from Africa, *R.a.SA* Riccardia amazonica collected from South America, *R.ob* Riccardia obtusa, *R.co* Riccardia compacta, *ns* non-significance

Significant codes *** 0.001, ** 0.01, * 0.05, . 0.1

**Fig. 8:**
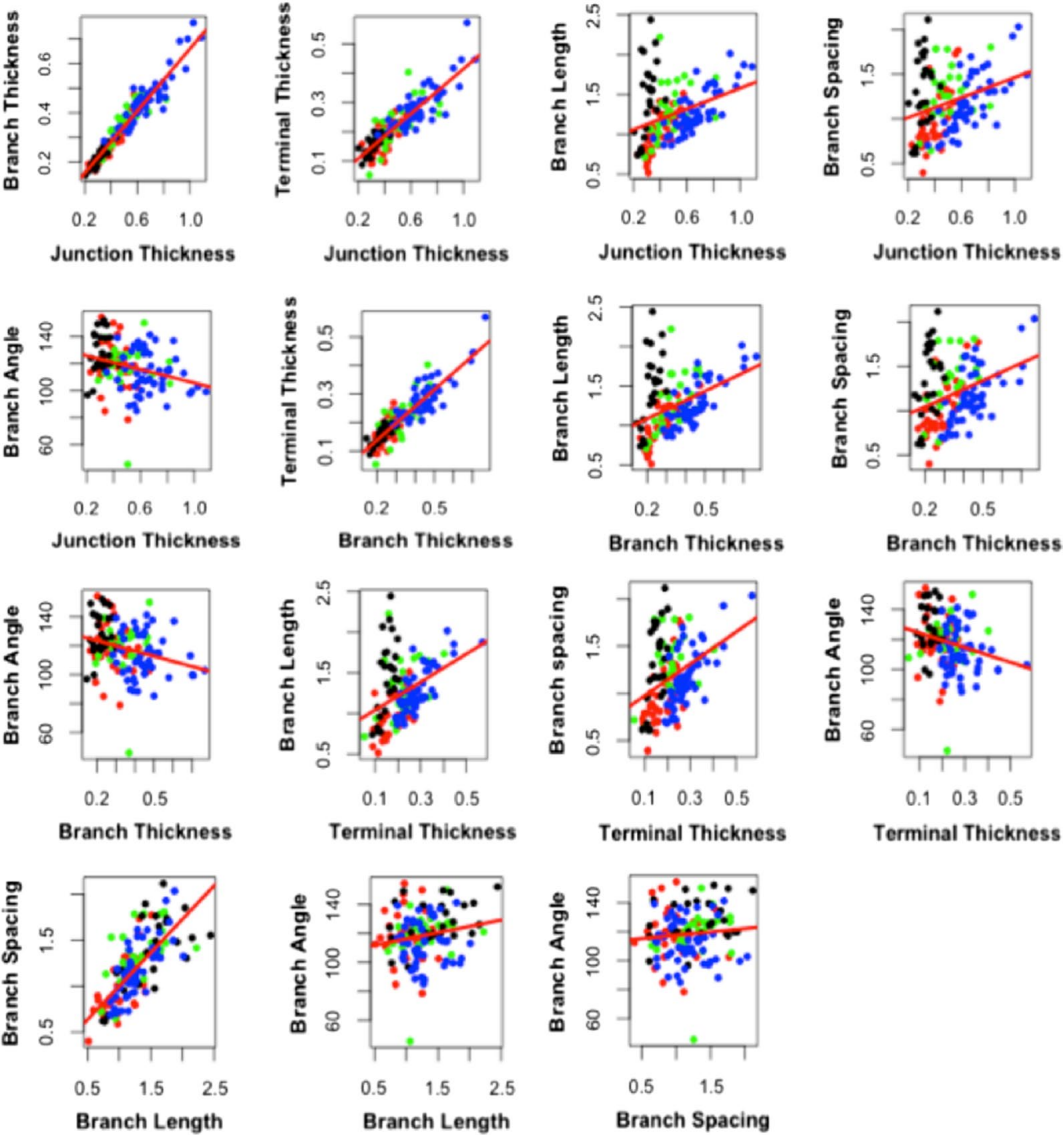
Bivariate plot of the six measurement variables (junction thickness, branch thickness, terminal thickness, branch length, branch spacing, and branch angle) with regression line (*red line*) of the four groups (*Riccardia amazonica* collected from Africa (*red*), *Riccardia amazonica* collected from South America (*green*), *Riccardia compacta* (*black*), and *Riccardia obtusa* (*blue*)

The correlation of these morphological variables provides some indication of how much each variable can contribute to the analysis. If two variables are very highly correlated, then they will be contributing shared information to the analysis. A Pearson product-moment correlation coefficient was computed to assess the relationship between two morphological variables. The bivariate plot (Fig. 8) shows the result of how the data spread among different groups by considering two out of the six morphological variables.

Figure 8 shows relationship between each pair of variables. It appears that all the widths given by the disc diameter measurements (da, db, and dc) are positively correlated to branch length and branch spacing. Considering *R. compacta* (black dots), its low variation of the disc diameter (da, db, dc) is associated with large variation of branch length and branch spacing. The other groups show almost linear relation between widths and branch length and branch spacing except *R. amazonica* SA, for which it is difficult to conclude on the relation between width and branch length. However, branch angle does not show clear relations with the other variables. Also, the relationship can be confirmed by the correlation coefficients (r) matrix with its corresponding *p* values (Table 3).

**Table 3:** Correlation coefficients between the measured variables and its corresponding *p* values

Morphological variable	da	db	dc	bl	bs	ba
da	1.00	0.96 (*)	0.87 (*)	0.34 (*)	0.29	−0.28 (ns)
db	0.96 (*)	1.00	0.89 (*)	0.40 (*)	0.33 (*)	−0.27 (ns)
dc	0.87 (*)	0.89 (*)	1.00	0.39 (*)	0.38 (*)	−0.24 (ns)
bl	0.34 (*)	0.40 (*)	0.39 (*)	1.00	0.72 (*)	0.19 (ns)
bs	0.29 (ns)	0.33 (*)	0.38 (*)	0.72 (*)	1.00	0.10 (ns)
ba	−0.28 (ns)	−0.27 (ns)	−0.24 (ns)	0.19 (ns)	0.10 (ns)	1.00

*ns* non-significance

* Significance at *p* value <0.0001

Amongst these correlated variables and their corresponding p-values Table 3, we found the four strongest significantly (*p* < 0.0001) correlated variables among the four groups. The strongest linear correlation (*r* = 0.96, *p* < 0.0001) was observed between junction thickness (da) and branch thickness (db). The other two strong correlations were between branch thickness (db) and terminal thickness (dc) (*r* = 0.89, *p* < 0.0001) and correlation (*r* = 0.87, *p* < 0.0001) between junction thickness (da) and terminal thickness (dc). Additionally, branch length (bl) is also correlated (*r* = 0.72, *p* < 0.0001) with branch spacing (bs).

We use canonical discriminant analysis to distinguish groups by considering the morphological measurements as the discriminating variables (MANOVA result shows significant differences with Wilks = 0.232 and *p* value <2.2e−16). Discriminant function analysis allows to find functions which are a combination of the variables to maximize the differences among the groups. From our samples, we obtained three discriminant functions (Table 4)

**Table 4:** Summary of canonical discriminant functions

Discriminant function	Canonical correlation	Eigen value	Wilks’s Lambda	Proportion	F-test	Num DF	*p* value
1	0.702	2.358	0.23	89.72	29.38	9	<2.20E−16 (***)
2	0.179	0.218	0.78	8.30	8.78	4	1.13E−06 (***)
3	0.049	0.052	0.95	1.98	6.97	1	0.0093 (**)

*Significant codes* *** 0.001, ** 0.01

As seen in Table 4, there are three discriminant functions and all of them are statistically significant. The percentage separation achieved by the first discriminant function and the second discriminant function account for 89.72 and 8.3% of the total variance existing in discriminating variables among the species, respectively. Therefore, the first discriminant function does achieve a good separation between the four groups, but the second discriminant function may improve the separation of the groups, so is it worth using the second discriminant function as well. For first discriminant function, its canonical correlation is high (0.702) which indicates good discrimination among the groups and its Wilks’s Lambda is low (0.23) showing that group means appear to differ with low p-value (<2.20E−16) which indicates the difference is significant, whereas other two discriminant functions are less correlated. The correlation between each variable and the discriminant functions can be revealed by the coefficients of discriminant function (Table 5).

**Table 5:** Discriminant function coefficients (a) and group means (b)

Morphological variable	Discriminant coefficients		
	Discriminant function 1	Discriminant function 2	Discriminant function 3
a			
Junction thickness	0.504	−0.196	1.128
Branch thickness	0.756	0.046	−1.320
Terminal thickness	0.086	0.079	0.163
Branch length	−0.677	−0.777	1.072
Branch spacing	−0.319	−0.246	−1.196
Branch angle	−0.002	0.071	0.0249

Species	Group mean		
	Discriminant function 1	Discriminant function 2	Discriminant function 3
b			
R_amazonica_AF	−0.383	0.749	0.026
R_amazonica_SA	−0.288	−0.229	−0.450
R_compacta	−2.548	−0.433	0.200
R_obtusa	1.708	−0.218	0.114

**Fig. 9:**
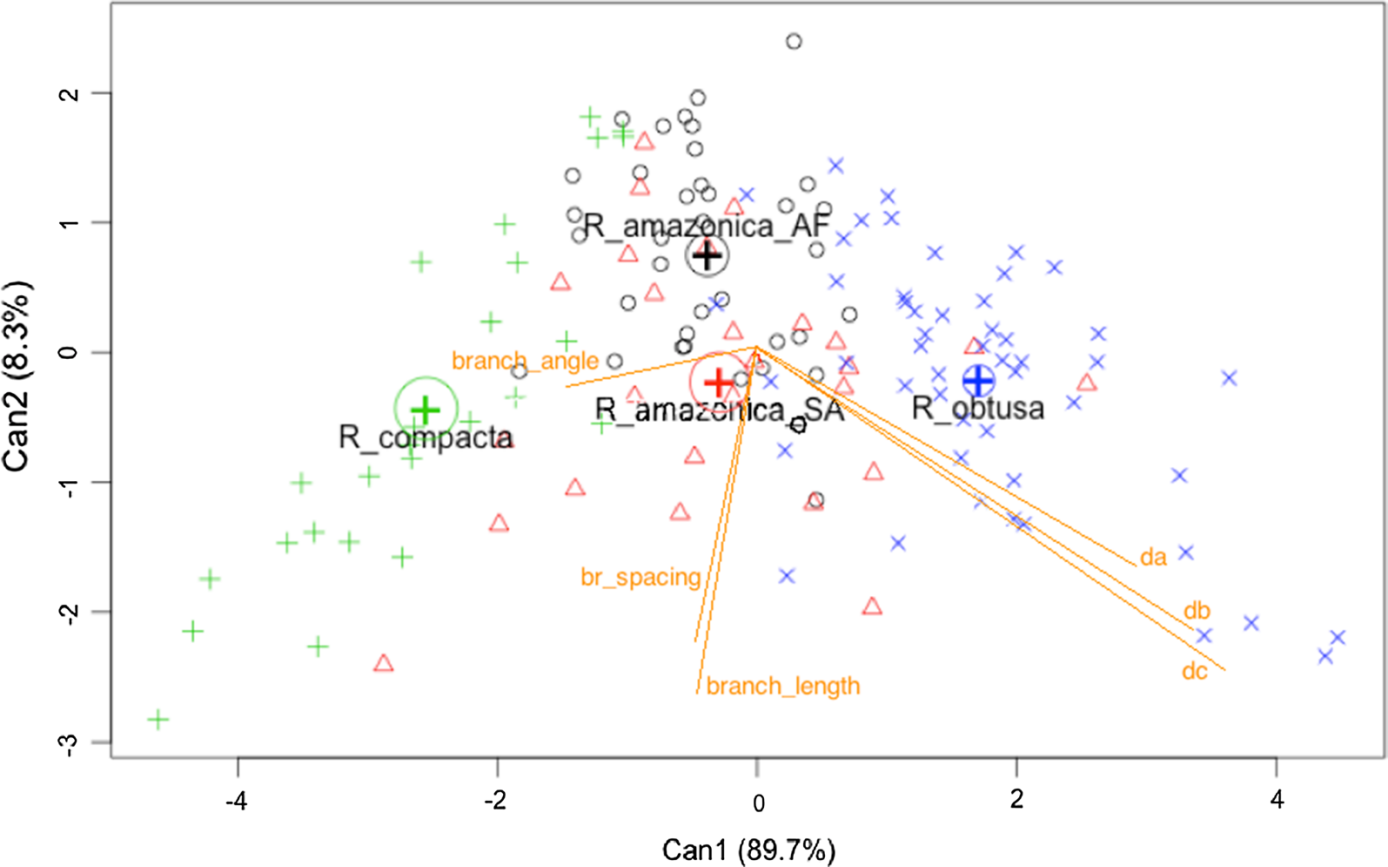
The scatterplot of the canonical discriminant function

From Table 5a, the first discriminant function is a linear combination of variables: 0.504*junction thickness + 0.756*branch thickness + 0.086*terminal thickness − 0.677*branch length − 0.319*branch spacing −0.002*branch angle. The coefficient values of the discriminant functions indicate that terminal thickness and branch angle have very little discriminating ability for these four groups. Junction thickness, branch thickness, branch length and branch spacing have a strong impact on the first discriminant function. The group means on the canonical variables (Table 5b) give some indication of how the groups are separated. The means on the first function show that R_compacta group separated farthest from the R_obtusa group. Interestingly by its mean, the second discriminant function is able to separate R_amazonica_AF from R_amazonica_SA, while the first discriminant function cannot. The best two discriminant functions are also virtualized by scatterplot (Fig. 9) to see how well the groups are separated.

Figure 9 shows that of the two canonical variables Can1 is able to clearly distinguish between *R. compacta* and *R. obtusa.* It can also separate *R. amazonica*_AF and *R. amazonica*_SA from *R. compacta* and *R. obtusa* with small overlap. However, Can1 cannot be used to separate *R. amazonica*_AF from *R. amazonica*_SA. In this case Can2 can be helpful, but with substantial overlap. Therefore, to achieve a good separation of the four groups, it would be best to use both the first and second discriminant functions together, since the first discriminant function can separate *R. compacta* and *R. obtusa* very well, and the second discriminant function can separate *R. amazonica*_AF and *R. amazonica*_SA.

Finally the discriminant function analysis is validated by classifying the samples to their original groups. As shown in Table 6, 83.8% (31/37) of *R. amazonica*_AF, 26.9% (7/26) of *R. amazonica*_SA, 68% (17/25) of *R. compacta*, and 84% (42/50) of *R. obtusa* are correctly classified. Therefore the proportions of all samples correctly classified are 70.3% (97/138).

**Table 6:** The classification matrix of the four groups as a result of canonical discriminant analysis

Species	N	Classified as			
		*R. amazonica AF*	*R. amazonica SA*	*R. compacta*	*R. obtusa*
*R. amazonica_AF*	*37*	31 (83.8%)	4 (10.8%)	1 (2.7%)	1 (2.7%)
*R. amazonica_SA*	*26*	8 (30.8%)	7 (26.9%)	4 (15.4%)	7 (26.9%)
*R. compacta*	*25*	7 (20%)	1 (4%)	17 (68%)	0 (0%)
*R. obtusa*	*50*	6 (12%)	2 (4%)	0 (0%)	42 (84%)

The *R. amazonica* samples collected from South America and Africa were supposed to belong to the same species. From Table 2, the ANOVA analysis shows a discrimination between the two groups (*R. amazonica*_AF vs *R. amazonica*_SA) as considering five measurements (da, db, dc, bl, and bs) except ba which is not significant. CDA is also applied to this case. The CDA result shows samples correctly classified to their original groups were 81% (Table 7).

**Table 7:** The classification matrix of *R. amazonica* from Africa and South America as a result of canonical discriminant analysis

Species	Classified as	
	*R. amazonica AF*	*R. amazonica SA*
*R. amazonica AF*	32 (86.5%)	5 (13.5%)
*R. amazonica SA*	7 (26.9%)	19 (73.1%)

## Discussion

We develop a semi-automated software to quantify some important morphological characters of liverworts which represent irregular and complex-shaped branching organisms. The characters are used for the purpose of species discrimination in genus *Riccardia*.

In our software, we use 2D image analysis techniques to automatically quantify the morphological variables. Most measurements are performed automatically. Some manual operations may still be required, such as removal of spurious branches that are created during the skeletonization due to boundary irregularities on the object in the image and loops in the skeleton which arise from overlapping branches. For these loops, automatic loop breaking is very complicated due to difficulty in deciding which edge in the loop should be deleted. Its complication is varied according to the number of loops which can lead to high possibility to produce false measurement and analysis. The software can report the number of loops as well as their locations, however, user has to decide how to do with the loops, which can be (1) manually removing an edge forming the loop, (2) edit but preserve the main characteristics of original sample image as much as possible by inserting a single pixel-wide gap to separate the overlapping branch or (3) prepare samples without loop. For our experiment, we have done (2) in order to compute the measurement automatically. For some thalli images presenting several overlapping branches, these branches were manually separated into new images without overlapping. Two or three images can be generated from the original and treated separately by the software. The original image with overlapping branches was also measured in order to compare the data.

The quantitative data of some morphological characters of African *R. amazonica*, *R. compacta*, and *R. obtusa* from literature were reported (Table 8). At the species level, it seems difficult to compare the literature data in Table 8 with our data due to the imprecision of measurements and different protocols. However, if we just compare width (junction thickness) and length (branch length), our data (Table 1) seem to provide lower values than the literature data. This could be due to the measurements between skeleton nodes that are not taken in account in the literature data, unlike our methods which defined precisely how to measure them based on points defined by junctions or nodes in the image skeleton.

**Table 8:** Comparison of quantitative data using different morphological characters among the three species of African *Riccardia*

Measurement	Species			References
	*R. amazonica (AF)*	*R. compacta*	*R. obtusa*	
Main axis width	500–800 µm	No data	Up to 900 µm	Perold [21–23]
Main axis length	13–14 mm	Up to 15 mm	10–15 mm	
Terminal branch length	575–875 µm	No data	350–2375 µm (no differences between primary and terminal)	
Terminal branch width	285–500 µm	No data	Up to 525 µm	
Angle between branches	No data	Up to 30°	40–70°	
Branch width	No data	No data	200-600 µm	Jones [18, 39] did not make difference between the axis levels
Branch length	No data	No data	0.6–1.5 (mm)	
Plant length	No data	Up to 20 mm long	No data	Meenks [19]
Plant length	No data	Up to 7 mm	No data	Arnell [16]
Plant width	No data	1–2 mm	No data	
Primary branch width	No data	Up to 500 µm	No data	

Classification rate from discriminant analysis showed that the species can be discriminated with 70.3% accuracy using the six morphological characteristics. The rate indicates the significance of morphological traits in the discrimination of *Riccardia* species. This result may confirm the indication that genetical differences could be expressed in the general dimensions of the thalli. The *R. amazonica* samples collected from South America and Africa were supposed to belong to the same species. With a classical morpho-anatomical revision of *R. amazonica,* a study of the historical material and recent collections [40] showed that some doubts remained on the inclusion of South American and African material in the same species. Therefore, we also investigated the two groups based on the morphological measurements. The ANOVA analysis shows a discrimination between the two groups and the CDA result shows samples correctly classified (Table 7) to their original groups were 81%.

We suggest, from our samples, that *R. amazonica* from South America and Africa show the significant differences in their morphometric features, and we propose the hypothesis that they could belong to different species. This hypothesis of species should be included in the revision of integrative taxonomy, which is the most consensual framework of today taxonomists [41–44]. It is engaged on the genus *Riccardia* in Africa, including both molecular and morphological analysis [25]. Morphological analysis is probably not as powerful as molecular analysis to delineate species because phenotype can be influenced by both original genotype and environmental conditions. However, this approach can be used as supplementary tool combined with other approach, such as molecular delineation methods (automatic barcode gap discovery (ABGD), generalized mixed yule coalescent (GYMC), haplowebs, see [44]). In case of congruence of the different results morphometry can support species hypothesis. On the opposite side, if the morphometric results separate samples that are recognized as the same species by other methods, it could suggest some other directions of investigation, for example, among ecological conditions to explain these differences. The morphological approach can also allow clear interspecific variations analysis. However, morphological variation together with molecular analysis and biogeographic studies are the best efficient way to classify species more accurately.

## Conclusions

A framework and software for taxonomic study using morphometric approach on 2D image have been presented in this paper. Our results provided evidence that quantitative characteristics determined by image processing and analysis techniques used in our software can be useful for taxonomic differentiation of the genus *Riccardia*. Also the characteristics are valuable for discriminating same species influenced by surrounding conditions from different geographical locations (*R. amazonica* collected from South America and Africa). Furthermore, our morphometric software can be applied to quantify branching growth form of other modular organisms.

## Additional files

**Additional file 1: Table SI1.** The origin of thalloid liverworts *Riccardia* collections. **Table SI2.** The number of *Riccardia* samples from different locations. **Table SI3.** P-value of Shapiro-Wilks normality test of each morphological variable among the four groups. **Table SI4.** Summary of ANOVA result of each morphological variable among all samples.

**Additional file 2: Figure SI1**. Measurements of sample images of species *Riccarida amazonica African* group (Row No. 1, 2*), Riccardia amazonica South-American* group (Row No. 3, 4), *Riccardia compacta* (Row No. 5, 6), and *Riccardia obtusa* (Row No. 7, 8). (a) Original binary image. (b) Skeleton (green), Junctions (pink), Terminals (blue). (c) Contour (light blue). (d) Junction thickness (red), Branch thickness (gray), (e) Branch length. (f) Terminal thickness (green), Branch spacing (white).

## Abbreviations

ANOVA: analysis of variance
ABGD: automatic barcode gap discovery
CDA: canonical discriminant analysis
CITES: convention on international trade in endangered species of wild fauna and flora
GNU GPL: GNU general public license
GYMC: generalized mixed yule coalescent
MANOVA: multivariate analysis of variance
RSA: root system architecture
