# Supplementary material for: Quantitative morphological analysis of 2D images of complex-shaped branching biological growth forms: the example of branching thalli of liverworts
Source: BMC Res Notes. 2017 Feb 20;10:103. doi: 10.1186/s13104-017-2424-0 (PMC5322791; doi:10.1186/s13104-017-2424-0)
Supplement: Supplementary file 1 — Additional file 1: Table SI1. The origin of thalloid liverworts Riccardia collections. Table SI2. The number of Riccardia samples from different locations. Table SI3. P-value of Shapiro-Wilks normality test of each morphological variable among the four groups. Table SI4. Summary of ANOVA result of each morphological variable among all samples. [file 13104_2017_2424_MOESM1_ESM.docx]

**Table SI1.** The origin of thalloid liverworts *Riccardia* collections.

| **Species** | **Collection number - Herbarium BarCode - Specimen information** | **Origin** |
| --- | --- | --- |
| ***Riccardia amazonica*** | CC1675-Emu71179-South America, Brazil, Rio negro  CC1679-EMu71223-EMu71234-South America, Brazil, Rio negro  CC1692-EMu74033-South America, Brazil, Rio negro | Herbarium Loan (MANCH- N° 2011.19) The University of Manchester Herbarium England, United Kingdom. |
|  | SIL424-UFP70053-South America, Brazil, Guaraibas | Herbarium Loan (UFP) Universidade federal de Pernambuco Herbarium, Brasil. |
|  | 027087-G00067578-Africa, Congo  1429a- G00264116-Africa, Sao Tomé  9668c- G00264170-Africa, La Réunion | Herbarium Loan (G-N°20110056C) Geneva Herbarium, Switzerland |
|  | PC101735-Africa, Sao Tomé | Herbarium Loan (PC) Paris France Cryptogamie |
|  | 1339, 1361C-Africa  U43460-Africa, Uganda | Herbarium Loan (EGER) Cryptogamic Herbarium Botanical Department of Eszterhàzy Karoly College. Hungary |
| ***Riccardia compacta*** | B20511-Africa, South Africa | Herbarium Loan (S- N° S-KBO 2011:58) Naturhistorika rikmuseet. Kryptogamnoatik, Stockolm, Sweden |
|  | 2884-Africa  25497-Africa | Herbarium Loan EGER) Cryptogamic Herbarium Botanical Department of Eszterhàzy Karoly College. Hungary |
| ***Riccardia obtusa*** | B20190, B20192-Africa, South Africa | Herbarium Loan (S- N° S-KBO 2011:58) Naturhistorika rikmuseet. Kryptogambotanik, Stockolm, Sweden |
|  | SN-E0043039- Nigeria (under the name of *R. limbata*)  6323-E00430415-Africa, Tanzania  M7409a-E00430479-Africa, Malawi  M8034a-E00430476-Africa, Malawi  U8053b-E00430486-Africa, Uganda | Herbarium Loan (E- N° 6534) Royal Botanical Garden Edinburgh, Scotland UK |

**Table SI2**. The number of *Riccardia* samples from different locations

| **Species** | **Specimen No.** | **Country** | **Herbarium** | **Full Name of Herbarium** | **Amount** |
| --- | --- | --- | --- | --- | --- |
| *Riccardia amazonica* AF | 1361C | GHA | G | Geneva Herbarium, Switzerland | 11 |
|  | 9668C | REU | G | Geneva Herbarium, Switzerland | 9 |
|  | EM74033 | CAM | MANCH | The University of Manchester Herbarium | 4 |
|  | G00264116 | NGR | G | Geneva Herbarium, Switzerland | 1 |
|  | G0067578 | COG | G | Geneva Herbarium, Switzerland | 1 |
|  | PC101735 | COG | PC | Paris Cryptogamie | 4 |
|  | U43460 | UGA | EGER | Cryptogamic Herbarium Botanical Department of Eszterhàzy Karoly College. Hungary | 5 |
|  | 1339c | GHA | G | Geneva Herbarium, Switzerland | 2 |
| *Total* |  |  |  |  | 37 |
| *Riccardia amazonica* SA | CC1675 | BRA | MAN | The University of Manchester Herbarium England, United Kingdom. | 2 |
|  | CC1679 | BRA | MAN | The University of Manchester Herbarium England, United Kingdom. | 3 |
|  | CC1680 | BRA | MAN | The University of Manchester Herbarium England, United Kingdom. | 3 |
|  | CC1684 | BRA | MAN | The University of Manchester Herbarium England, United Kingdom. | 5 |
|  | CC1686 | BRA | MAN | The University of Manchester Herbarium England, United Kingdom. | 4 |
|  | EPX77 | BRA | UFP | Universidade federal de Pernambuco Herbarium, Brasil. | 6 |
|  | SIL424 | BRA | UFP | Universidade federal de Pernambuco Herbarium, Brasil. | 3 |
| *Total* |  |  |  |  | 26 |
| *Riccardia compacta* | CH2884 | SAF | S | Naturhistorika rikmuseet. Kryptogambotanik, Stockolm, Sweden | 7 |
|  | 25497 | GHA | G | Geneva Herbarium, Switzerland | 2 |
|  | TYP compacta | SAF | G | Geneva Herbarium, Switzerland | 16 |
| *Total* |  |  |  |  | 25 |
| *Riccardia*  *Obtusa* | 6323 | TZA | G | Geneva Herbarium, Switzerland | 15 |
|  | B20190 | SAF | S | Naturhistorika rikmuseet. Kryptogambotanik, Stockolm, Sweden | 2 |
|  | B20192 | SAF | S | Naturhistorika rikmuseet. Kryptogambotanik, Stockolm, Sweden | 16 |
|  | E00430393 | UGA | E | Royal Botanical Garden Edinburgh, Scotland UK | 3 |
|  | M7409a | MLW | EGER | Cryptogamic Herbarium Botanical Department of Eszterhàzy Karoly College. Hungary | 2 |
|  | M8034a | MLW | EGER | Cryptogamic Herbarium Botanical Department of Eszterhàzy Karoly College. Hungary | 6 |
|  | U8053b | UGA | EGER | Cryptogamic Herbarium Botanical Department of Eszterhàzy Karoly College. Hungary | 1 |
|  | B12531 | REU | S | Royal Botanical Garden Edinburgh, Scotland UK | 5 |
| *Total* |  |  |  |  | 50 |

**Table SI3.** P-value of Shapiro-Wilks normality test of each morphological variable among the four groups

| **Morphological**  **variable** | **R_amazonica_SA** | **R_amazonica_AF** | **R_compacta** | **R_obtusa** |
| --- | --- | --- | --- | --- |
| **da** | 0.028(*) | 0.2199(*) | 0.9285 (*) | 0. 1353(*) |
| **db** | 0.5332(*) | 0.1059 (*) | 0. 9402(*) | 0. 002(ns) |
| **dc** | 0. 7032(*) | 0. 0789(*) | 0. 6558(*) | 0. 0001(ns) |
| **bl** | 0.5042 (*) | 0. 6257(*) | 0. 5848(*) | 0. 0085(ns) |
| **bs** | 0. 3617(*) | 0. 0602(*) | 0. 302(*) | 0. 1477(*) |
| **ba** | 1.77e-05(ns) | 0. 2843(*) | 0. 116(*) | 0. 1778(*) |

*(*): significance level at 0.005, (ns): non-significance*

**Table SI4.** Summary of ANOVA result of each morphological variable among all samples.

| **Morphological**  **variable** | **F-value** | **p-value** |
| --- | --- | --- |
| **da** | *58.032* | *<2.2e-16(***)* |
| **db** | *53.378* | *<2.2e-16(***)* |
| **dc** | *33.712* | *2.669e-16(***)* |
| **bl** | *9.9518* | *5.741e-06(***)* |
| **bs** | *7.5955* | *9.935e-05(***)* |
| **ba** | *5.4596* | *0.001429(**)* |

*Signif. codes: 0 '***' 0.001 '**' 0.01 '*' 0.05 '.' 0.1 ' ' 1*
