# Supplementary material for: Quantitative morphological analysis of 2D images of complex-shaped branching biological growth forms: the example of branching thalli of liverworts
Source: BMC Res Notes. 2017 Feb 20;10:103. doi: 10.1186/s13104-017-2424-0 (PMC5322791; doi:10.1186/s13104-017-2424-0)
Supplement: Supplementary file 2 — Additional file 2: Figure SI1. Measurements of sample images of species Riccarida amazonica African group (Row No. 1, 2), Riccardia amazonica South-American group (Row No. 3, 4), Riccardia compacta (Row No. 5, 6), and Riccardia obtusa (Row No. 7, 8). (a) Original binary image. (b) Skeleton (green), Junctions (pink), Terminals (blue). (c) Contour (light blue). (d) Junction thickness (red), Branch thickness (gray), (e) Branch length. (f) Terminal thickness (green), Branch spacing (white). [file 13104_2017_2424_MOESM2_ESM.docx]

| 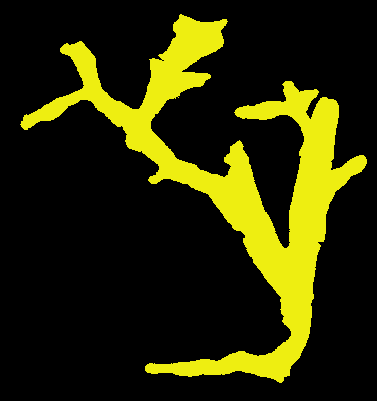  (1) | 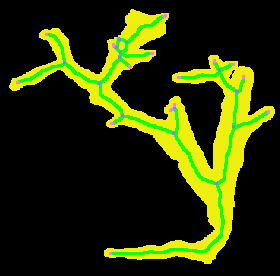 | 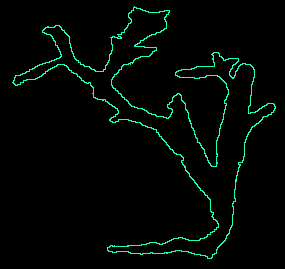 | 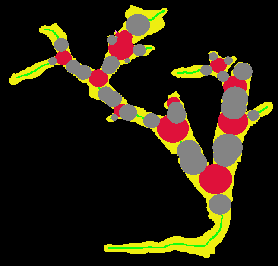 | 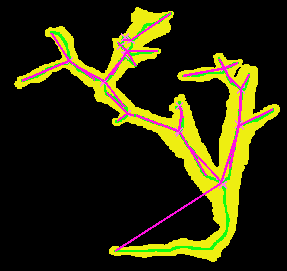 | 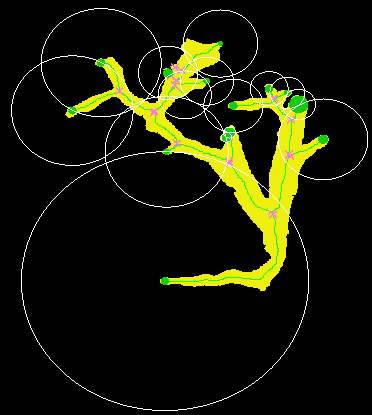 |
| --- | --- | --- | --- | --- | --- |
| 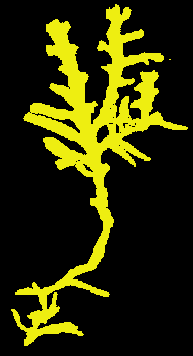  (2) | 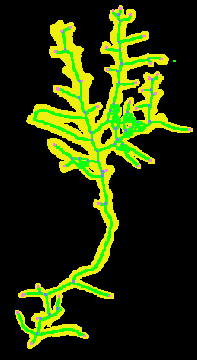 | 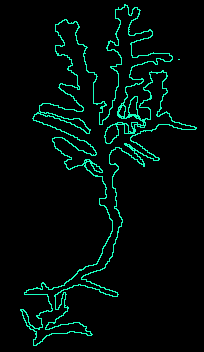 | 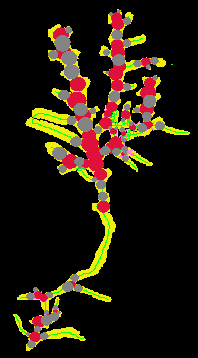 | 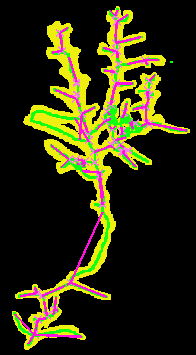 | 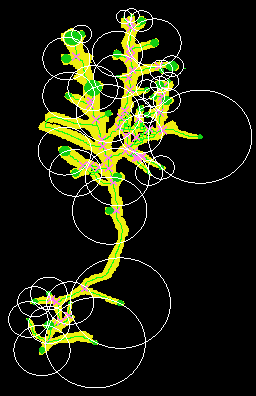 |
| 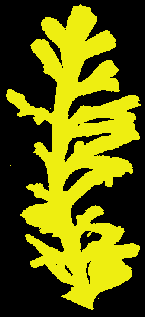  (3) | 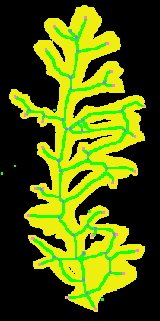 | 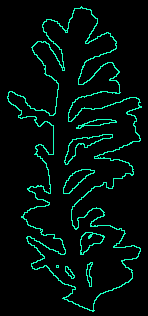 | 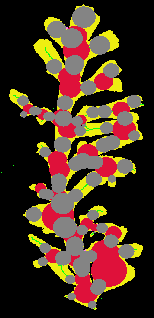 | 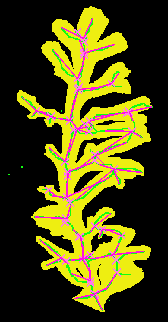 | 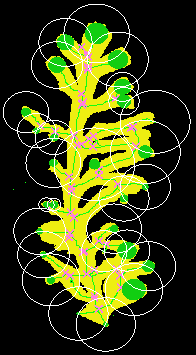 |
| 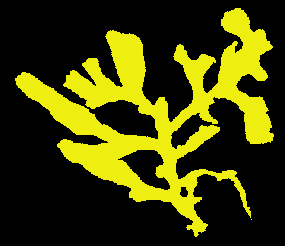  (4) | 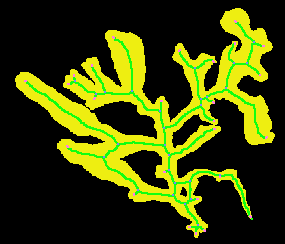 | 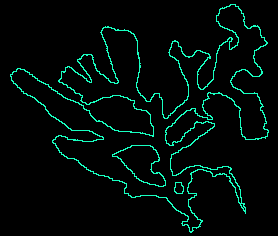 | 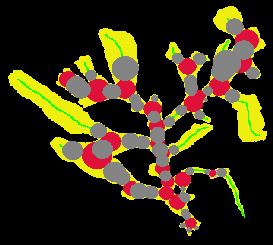 | 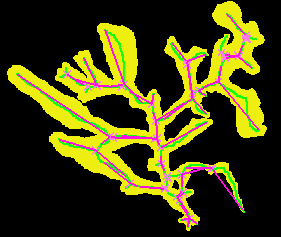 | 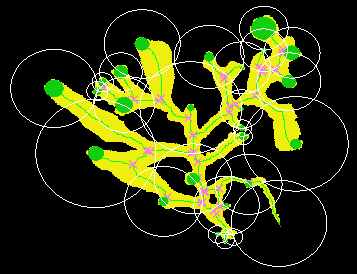 |
| 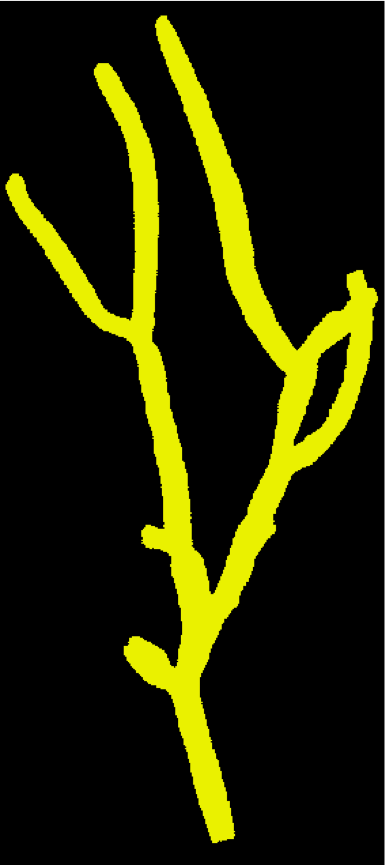  (5) | 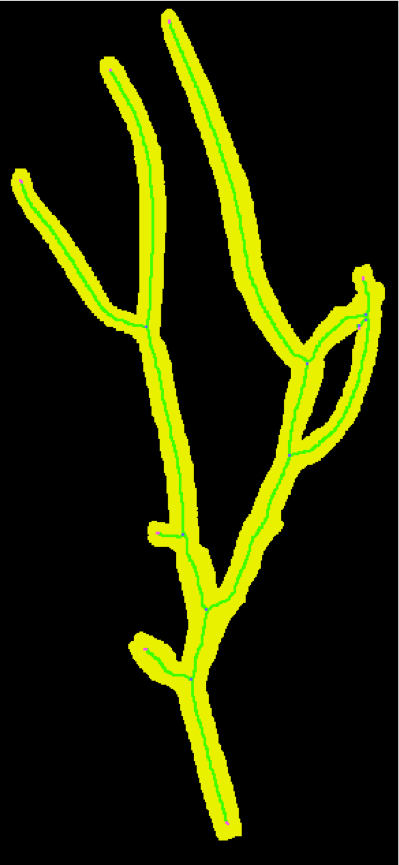 | 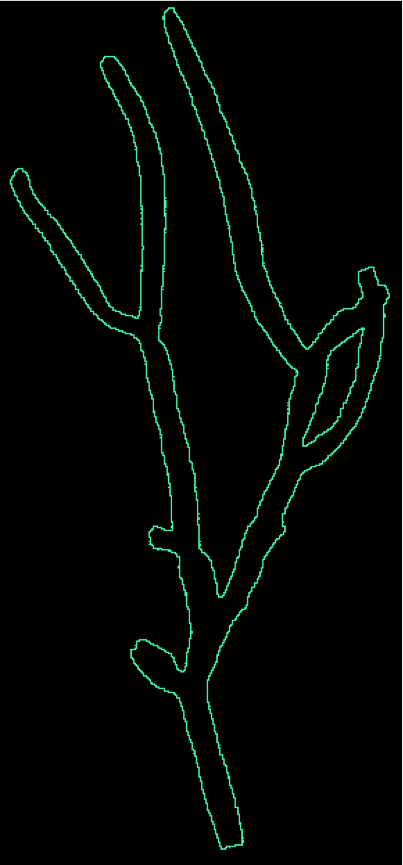 | 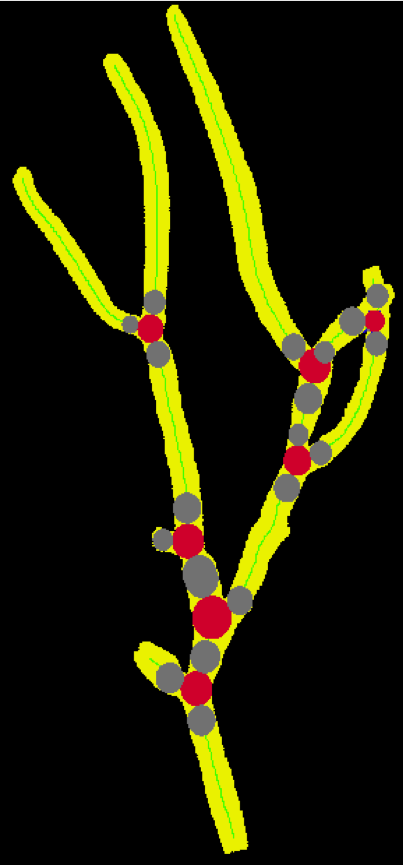 | 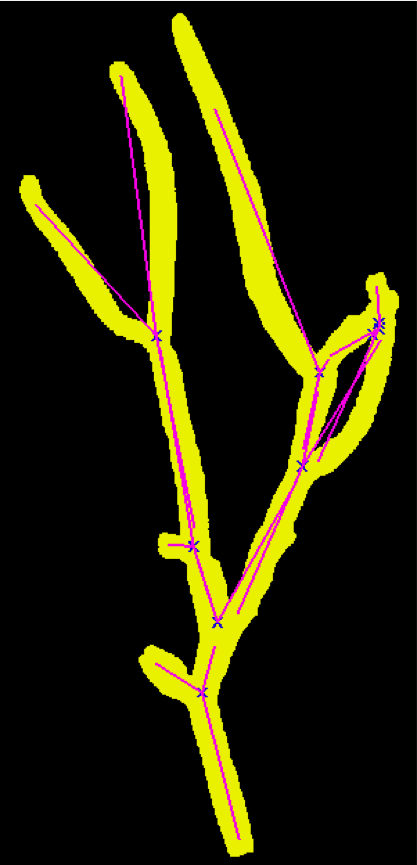 | 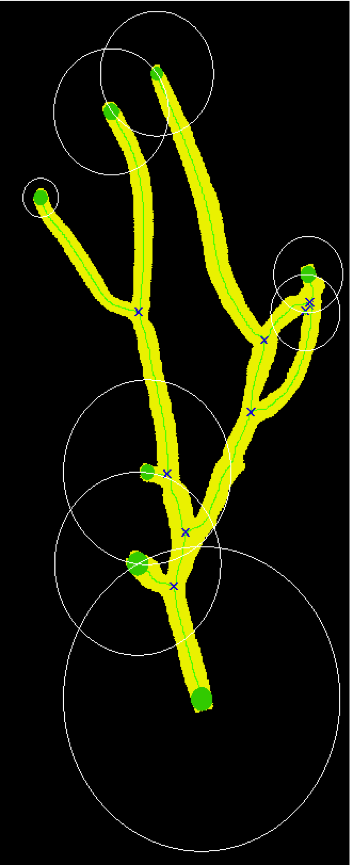 |
| 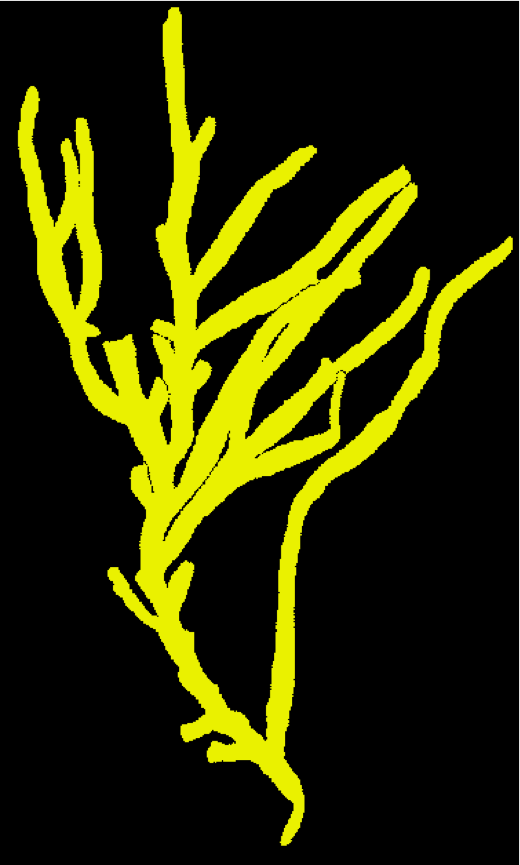  (6) | 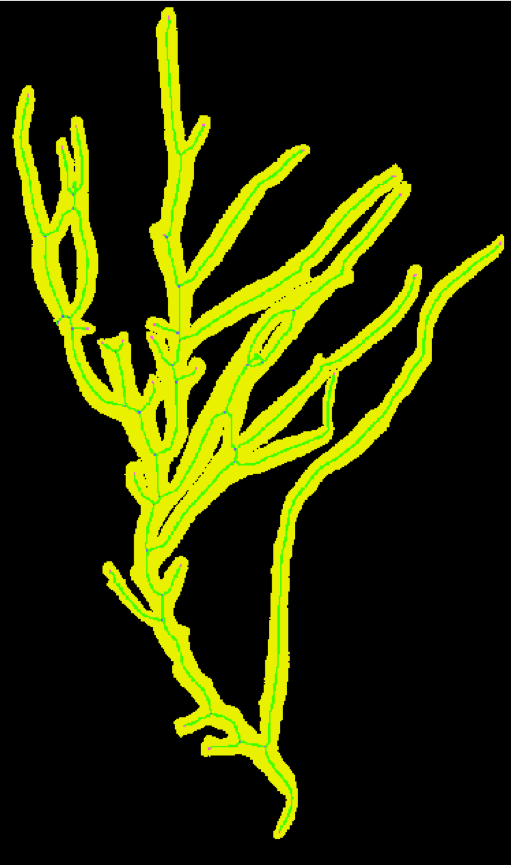 | 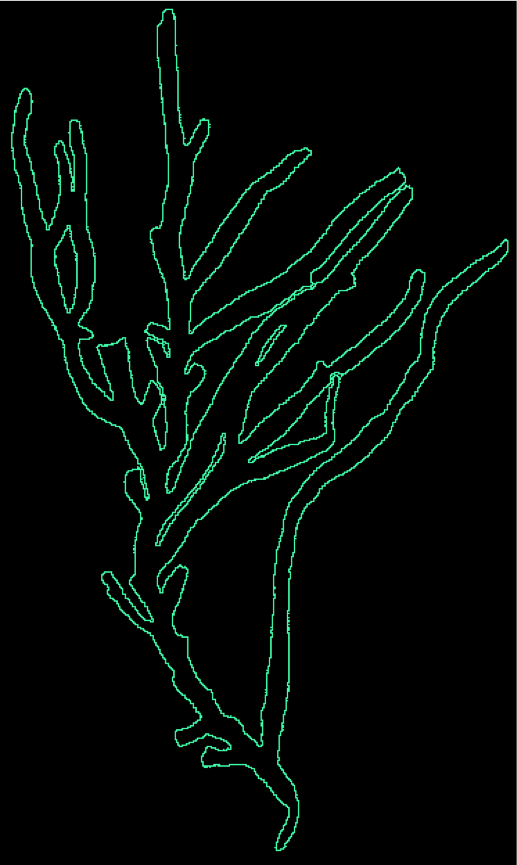 | 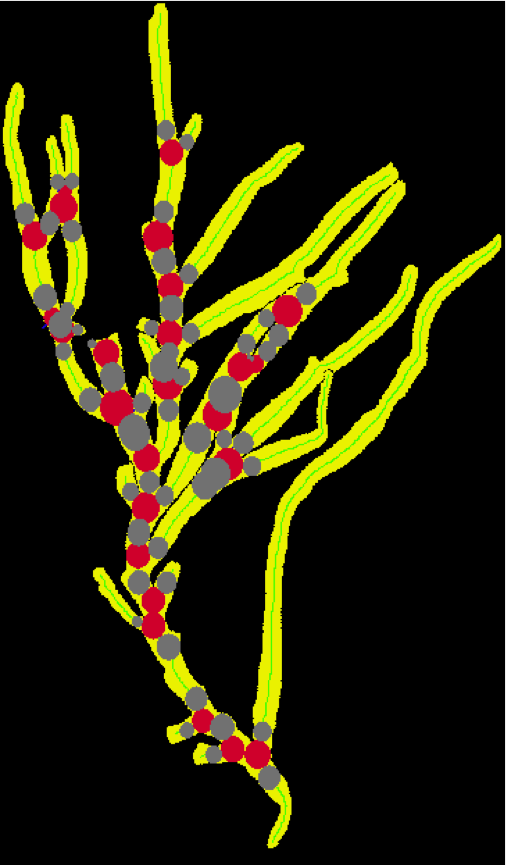 | 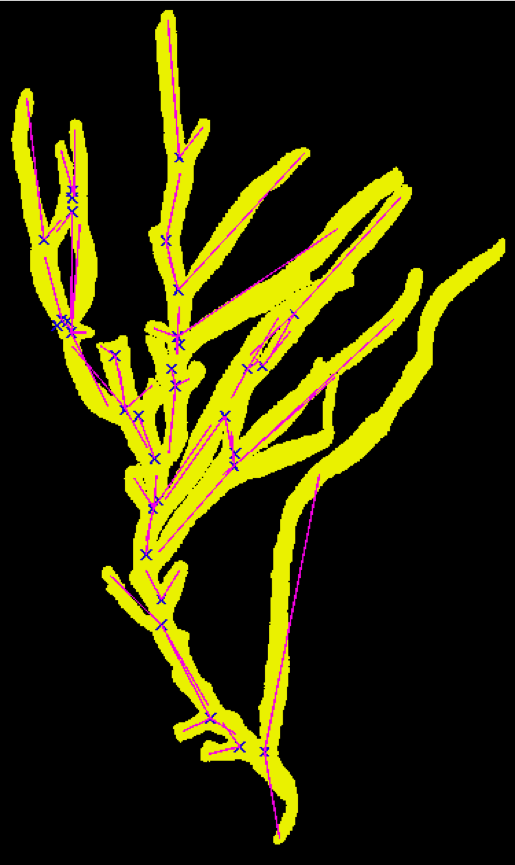 | 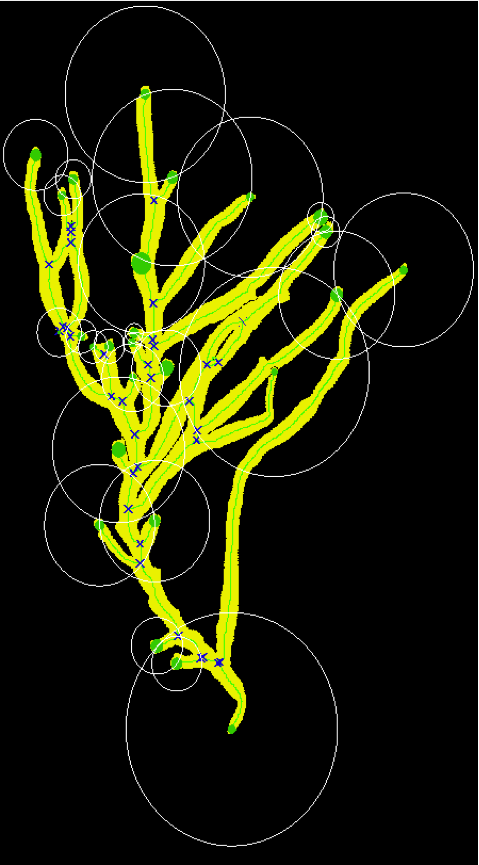 |
| 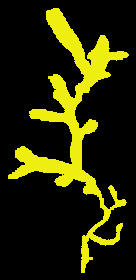  (7) | 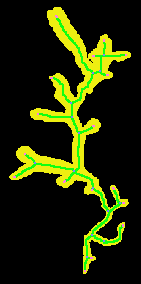 | 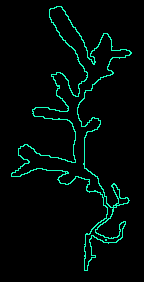 | 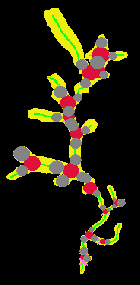 | 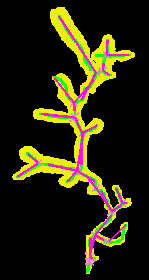 | 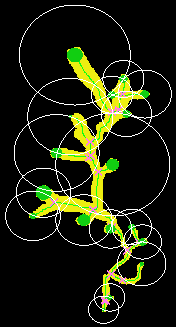 |
| 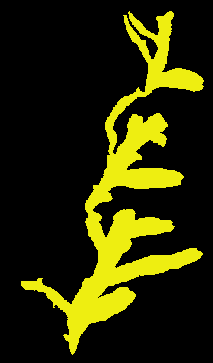  (8) | 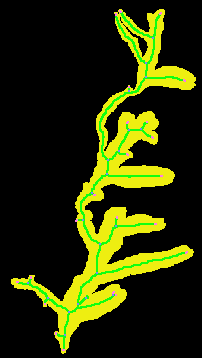 | 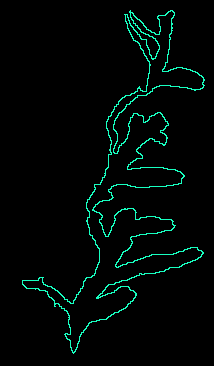 | 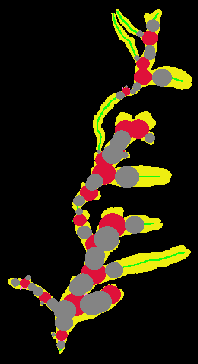 | 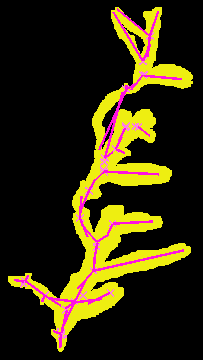 | 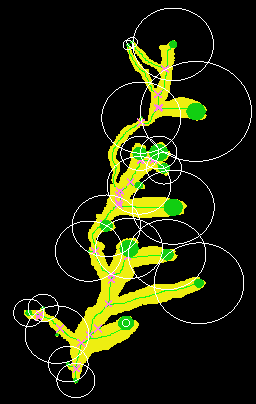 |
| (a) | (b) | (c) | (d) | (e) | (f) |

**Figure SI1.** Measurements of sample images of species *Riccardia amazonica African* group (Row No. 1, 2*), Riccardia amazonica* *South-American* group (Row No. 3, 4), *Riccardia compacta* (Row No. 5, 6), and *Riccardia* *obtusa* (Row No. 7, 8)**.** (a) Original binary image. (b) Skeleton (green), Junctions (pink), Terminals (blue). (c) Contour (light blue). (d) Junction thickness (red), Branch thickness (gray), (e) Branch length. (f) Terminal thickness (green), Branch spacing (white).
